# *Plasmodium* mono and mixed-infections in India: a tale of see-saw in species misidentification

**DOI:** 10.3389/fcimb.2025.1695062

**Published:** 2026-01-02

**Authors:** Nimita Deora, Veena Pande, Abhinav Sinha

**Affiliations:** 1 Department of Epidemiology, Indian Council of Medical Research (ICMR)-National Institute of Malaria Research, Dwarka, New Delhi, India; 2 Department of Biotechnology, Kumaun University, Nainital, Uttarakhand, India; 3 Academy of Scientific and Innovative Research, Ghaziabad, India

**Keywords:** diagnosis, malaria, microscopy, misidentification, mixed infection, PCR

## Abstract

**Introduction:**

Malaria is still a public health challenge across many regions of the world. With significant efforts, it has become a target for elimination in near future. However, elimination requires early and accurate diagnosis and despite the fact that malaria elimination is nearing in several countries, specific and sensitive diagnosis of malaria and its causative species remains inadequate in many near-elimination settings, including India. With the advent and increasing usage of nucleic acid-based detection of *Plasmodium* infection, the diagnostic limitations of microscopy, including errors in species identification, have become more prominent.

**Methods:**

The purpose of this study was to determine the magnitude of *Plasmodium* species misidentification by microscopy in India based on previously published reports that performed microscopy and PCR to the same samples. A total of 2706 microscopy-PCR pairs were extracted from 16 different locations across 11 Indian states. Region-specific and species-wise misidentification rates were also estimated.

**Results:**

The analyses revealed 15% misidentification rate (408/2706). Surprisingly, microscopy misidentified >98% of mixed-infections (400/405) as mono-infections (almost all as *P. falciparum* mono-infections). The study identifies Jharkhand and Madhya Pradesh as major contributors (>20%) to *Plasmodium* species misidentification by microscopy.

**Discussion:**

These findings suggest that we are overestimating *P. falciparum* burden, potentially wasting elimination resources, and underestimating non-falciparum species. The study also addresses an important issue concerning analysis of misidentification and sub-microscopic infection data. It proposes an analysis approach that is expected to help in deciphering misidentification and sub-microscopic infections in a more granular manner, generating actionable data for countries targeting malaria elimination.

## Graphical Abstract

**Figure:**
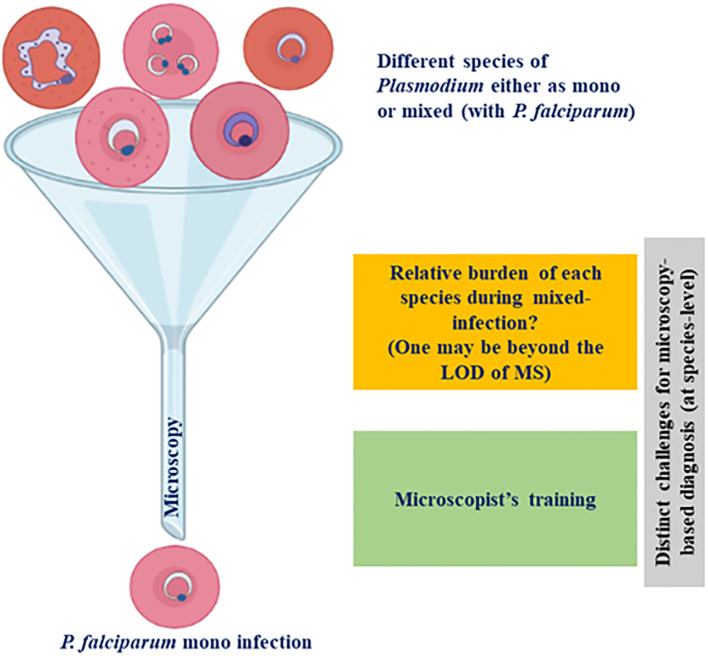
Possible challenges for microscopy at species-level diagnosis. The figure represents the difficulties for microscopy in diagnosing a non-falciparum species when it co-exists with *P. falciparum*.

## Introduction

Globally, 263 million people were estimated to have malaria with 0.6 million estimated deaths in 2023 (World malaria report 2024: addressing inequity in the global malaria response, 2024). The World Health Organization’s (WHO) South East Asia Region had only 1.5% of the estimated global burden, but India contributed half of these cases with *P. vivax* (Pv) and *P. falciparum* (Pf) as major species and negligible reporting of the non-Pv and non-Pf parasite species (World malaria report 2024: addressing inequity in the global malaria response, 2024). It is also to be noted that there have been discrepancies between the number of cases reported by National Malaria Control Program and that estimated by the WHO (Deora et al., 2025). Although 9 *Plasmodium* species are known to infect humans, Pf, Pv and *P. knowlesi* (Pk, to a lesser extent), are known to cause the most severe/fatal forms of malaria (WHO guidelines for malaria, 16 October 2023, 2023). Therefore, the current recommended antimalarial treatment in India is targeted for Pf (artemisinin-based combination therapy or ACTs) and Pv (chloroquine). Further, Pv and *P. ovale* (Po) are known for their hepatic dormant stages (hypnozoites) which may result in clinical relapses (WHO guidelines for malaria, 16 October 2023, 2023). Hence, in addition to chloroquine, a 14-day radical treatment with primaquine is recommended for Pv and Po, only after ensuring Glucose-6-phosphate dehydrogenase (G6PD) sufficiency. All mixed infections involving Pf are recommended to be treated as Pf with additional 14-day treatment with primaquine if the co-infecting species is Pv and/or Po (WHO guidelines for malaria, 16 October 2023, 2023).

The diagnostic modalities currently available for malaria include microscopy, rapid diagnostic tests (RDTs) and nucleic acid amplification tests (PCRs). The limit of detection (LoD) of microscopy is 10–100 parasites/μL of blood. In contrast, PCR-based methods have LoD between 0.022 (ultrasensitive PCR) and 5 (standard PCR) parasites/μL of blood (World Health Organization, 2017; World Health Organization, 2018; Chauhan and Sinha, 2021). Although bivalent RDTs are rapidly gaining popularity as PoC diagnostics, their lower sensitivity (LoD ~200 parasites/μL of blood) and impending HRP2 deletions in Pf, limit their use. On the other hand, PCRs are limited to research settings. Apart from lower costs, microscopy has distinct advantages of directly visualizing the parasites, identifying parasite species and stages, and quantifying parasite density – all at one step. In addition, microscopy, if performed correctly, may also identify certain human blood cell disorders and pathogens.

Differentiating *Plasmodium* spp. is challenging in malaria microscopy due to the close morphological similarity among certain species. For example, it is often difficult for inexperienced microscopists to distinguish Po from Pv (Kotepui et al., 2020; Calderaro et al., 2021) and *P. malariae* (Pm) from Pk (Kotepui et al., 2020; Calderaro et al., 2021; Mahittikorn et al., 2021) thus underestimating the real species-specific burden of *Plasmodium*. Microscopy being a skill-based technique (National Strategic Plan (NSP), [[NoYear]]; McKenzie et al., 2003; Maguire et al., 2006; Moura et al., 2014; World Health Organization, 2016), under-skilled microscopists often tend to mis-identify lesser-known *Plasmodium* spp. thus leading to their misdiagnosis, especially when present with Pf during mixed infections (Edson et al., 2010). In such cases, treatment is provided for Pf alone, whereas co-infecting species (Pv/Po) remain untreated for radical cure. Therefore, for a particular species, false-negative microscopy (misidentified as another species) could result in potentially serious consequences. Accurately distinguishing between Pf and non-Pf infections and identifying all *Plasmodium* spp. (present in a region) therefore, is a minimum competence required for microscopy (World Health Organization, 2016).

Compromised sensitivity (false negative/FN or sub-microscopic infections/SMIs) and specificity (false positive/FP or misidentified *Plasmodium* spp.) may be detrimental for countries aiming malaria elimination. Despite the gravity, targeted research on these issues has rarely been a priority and therefore the way FN/SMI data are analyzed and reported tends to be flawed. Deep understanding and analysis of microscopy and PCR paired data is therefore critical as most of the published reports interpret this data from flawed and crude analysis which may lead to incorrect conclusions.

In order to address the above concerns the current study attempts to depict the magnitude of *Plasmodium* spp. misidentification in India and to investigate whether misidentification of mixed infections is associated only with uncommon/rare *Plasmodium* spp. This manuscript also cautions and critiques ‘overall’ analysis and proposes a one-to-one analysis of microscopy-PCR paired data which is more factual and reveals much needed information for malaria control, globally. The suggested analysis is expected to reveal more granular and actionable information that would be immensely helpful for the National authorities driving malaria elimination in various countries.

## Methodology

Studies that were included for this study were selected from a database that had previously been created to assess the worldwide burden of mixed *Plasmodium* spp. infection. The databases were searched in April 2020 with no date-range filter. The study’s protocol was registered into the International Prospective Register of Systematic Reviews (PROSPERO) with the registration number CRD42021234278.

**Figure 1:**
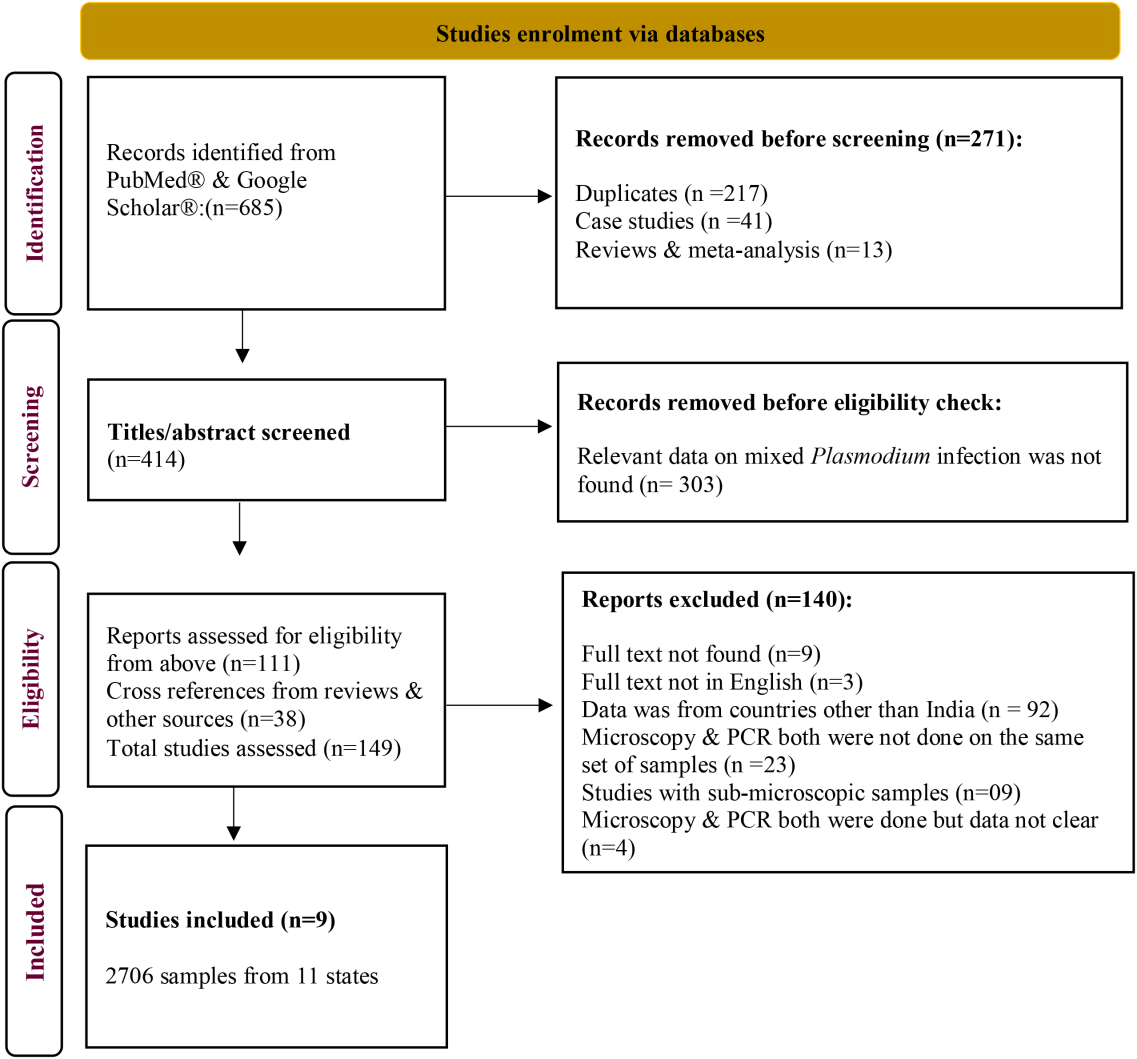
Study enrolment following PRISMA guidelines.

From the above database, studies that contained data from India were included if microscopy and PCR both were applied to the same set of samples for diagnosing *Plasmodium* spp. (**Figure 1**).

From the enrolled studies, the year of data-collection, geographical area, total number of samples tested, number of samples positive/negative for the presence of *Plasmodium* spp. by microscopy and PCR (concordant and discordant results) were recorded (**Supplementary Tables S1, S2**). Samples were analyzed by data collection sites and if the same data collection sites were found in numerous studies, they were not grouped together but were examined individually. Misidentified *Plasmodium* species (misdiagnosed by microscopy as a different *Plasmodium* species) from that detected by PCR were identified. Samples that were detected positive by microscopy but negative by PCR for all *Plasmodium* species were also categorized as misidentified.

All mixed *Plasmodium* species infections that were misidentified as mono-infection (by microscopy) of either species were also categorized under misidentification as it was uncertain if the other species (in a mixed-infection) was missed by microscopy because it was sub-microscopic or because the other one was detected sooner.

Burden of overall and species-specific *Plasmodium* misidentification was estimated as:

A. Overall and region-specific misidentification rate (%)


$$=\frac{\text{total misidentified cases by microscopy }(\text{irrespective of species})\;}{\text{total cases identified by microscopy}}*100$$

B. Species-specific misidentification rate (%) was estimated in three ways as below:


$$=\frac{\text{total misidentified cases by microscopy }(\text{of a particular species})\;}{\text{total cases identified by microscopy}}*100$$

$$=\frac{\text{total misidentified cases by microscopy }(\text{of a particular species})\;}{\text{total misidentified cases }(\text{irrespective of species})}*100$$

$$=\frac{\text{total misidentified cases by microscopy }(\text{of a particular species})\;}{\text{total PCR confirmed cases }(\text{of that particular species})}*100$$

## Results

**Figure 2:**
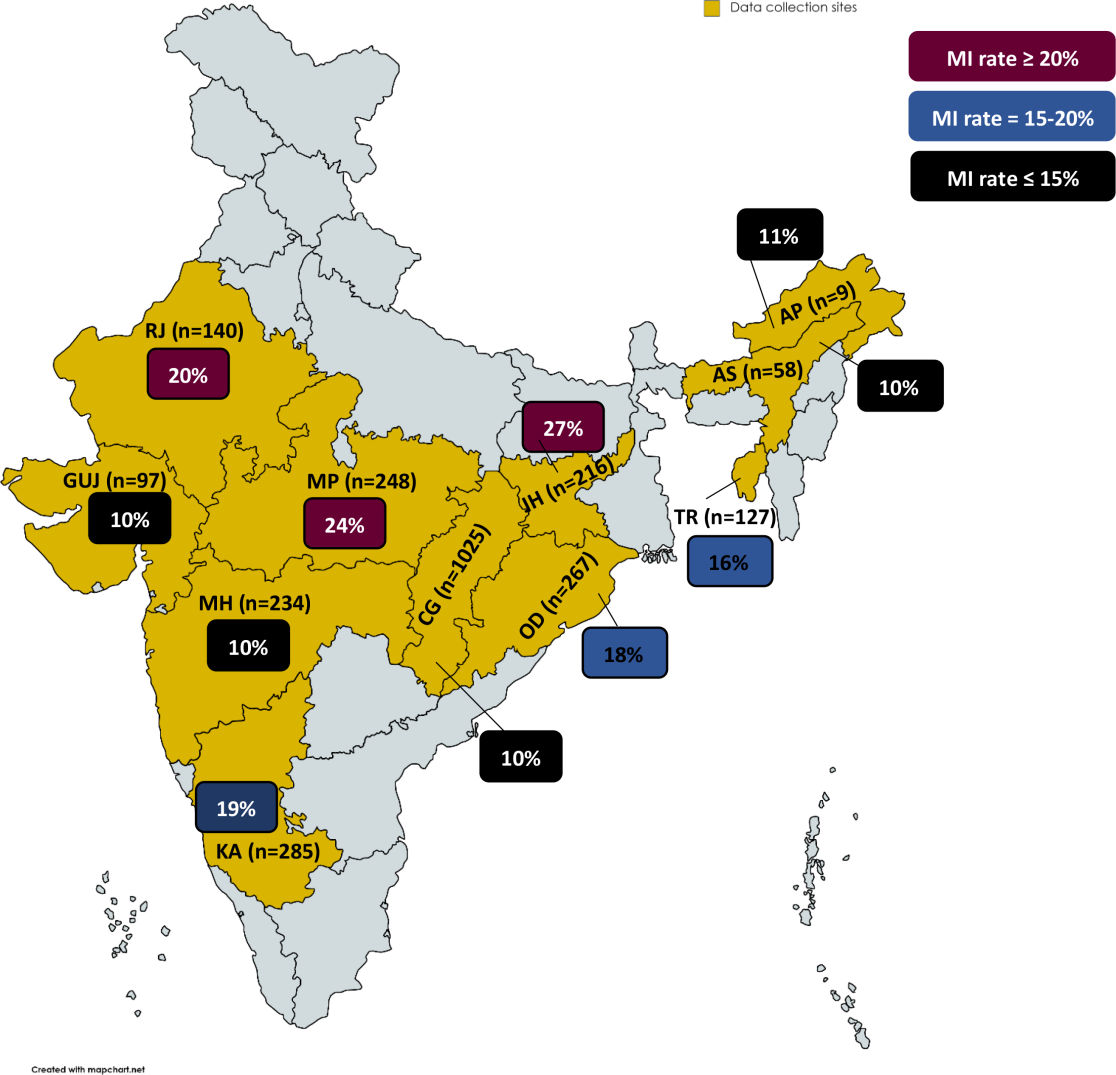
Geographical distribution of *Plasmodium* species misidentification (by microscopy). The figure depicts the contribution of various geographical sites (Indian states) to microscopy misidentification of *Plasmodium* species (calculated as formula given below). Here, the yellow shaded areas indicate the geographic locations from which data have been gathered. These areas are labelled with the state name initials and the number (n) of samples that have tested positive for *Plasmodium* (by microscopy). According to their contribution, the percentages of MI (from the total positive samples for *Plasmodium*) from that specific region have been mentioned in boxes and coloured burgundy (MI rate ≥20%), blue (MI rate = 15-20%), and black (MI rate ≤15%). Overall and region-specific misidentification rate (%) $=\frac{\text{total misidentified cases by microscopy }(\text{irrespective of species})\;}{\text{total cases identified by microscopy}}*100$. AP, Arunachal Pradesh; AS, Assam; CH, Chhattisgarh; GUJ, Gujarat; JH, Jharkhand; KA, Karnataka; MP, Madhya Pradesh; MH, Maharashtra; OD, Odisha; RJ, Rajasthan; TR, Tripura.

The study finally included 9 published records (Mohapatra et al., 2008; Bharti et al., 2013; Dhiman et al., 2013; Singh et al., 2013; Chaturvedi et al., 2015; Krishna et al., 2015; Rishikesh et al., 2015; Saravu et al., 2016; Krishna et al., 2017; Deora et al., 2022) (**Figure 1**) encompassing 2706 microscopy-positive *Plasmodium* samples collected between 2005 and 2015 from 11 states and 16 sites (**Supplementary Table S2**; **Figure 2**). All of the studies included in this analysis used a common nested PCR approach.

### Microscopy misidentification rates

Overall misidentification rate was 15% (408/2706) including negative samples (n=7; microscopy-positive but PCR-negative for all *Plasmodium* species). Further analyses revealed that all (99.75%; 400/401) misidentified samples were mixed-infections except one mono-infection, that is Pm (**Table 1**). Region-wise overall misidentification rates reveal that misidentification of *Plasmodium* species was highest in Jharkhand (27%; 58/216) followed by Madhya Pradesh (24%; 60/248), Rajasthan (20%; 28/140), Karnataka (19%; 53/285), Odisha (18%; 48/267) and Tripura (16%; 20/127). Misidentification rate in the remaining five regions (Arunachal Pradesh, Assam, Gujarat, Chhattisgarh, and Maharashtra) ranged from 10 to 11% (**Figure 2**).

**Table 1:** *Plasmodium* species misidentified by microscopy.

Species confirmed by nPCR (n)	Misidentified by microscopy; n (%)	Misidentified into; n (%)
Pf (2040)	0 (0)	NA
Pv (232)	0 (0)	NA
Pm (22)	1 (5)	Pf (100)
PfPv (355)	351 (99)	Pf (?) and Pv (?)
PfPm (29)	28 (97)	Pf (?), Pm (?) and PfPv (?)
PfPo (8)	8 (100)	Pf (100)
PvPm (3)	3 (100)	Pm (100)
PfPvPm (5)	5 (100)	Pf (100)
PfPvPo (3)	3 (100)	Pf (100)
PfPmPo (1)	1 (100)	Pf (100)
PfPvPoPm (1)	1 (100)	Pf (100)
All species (2699)	401 (15)	Pf (?), Pv (?), Pm (?) and PfPv (?)

The table shows the total number of *Plasmodium* species confirmed by nested PCR in 2699 samples for which microscopy was also performed. The frequency and percentage of misidentified species by microscopy and the species into which microscopy misidentified them is also shown. As the proportional breakup of species was not available from the contributing studies, the same is indicated by an interrogation (?) mark.

### Misidentified *Plasmodium* species

**Figure 3:**
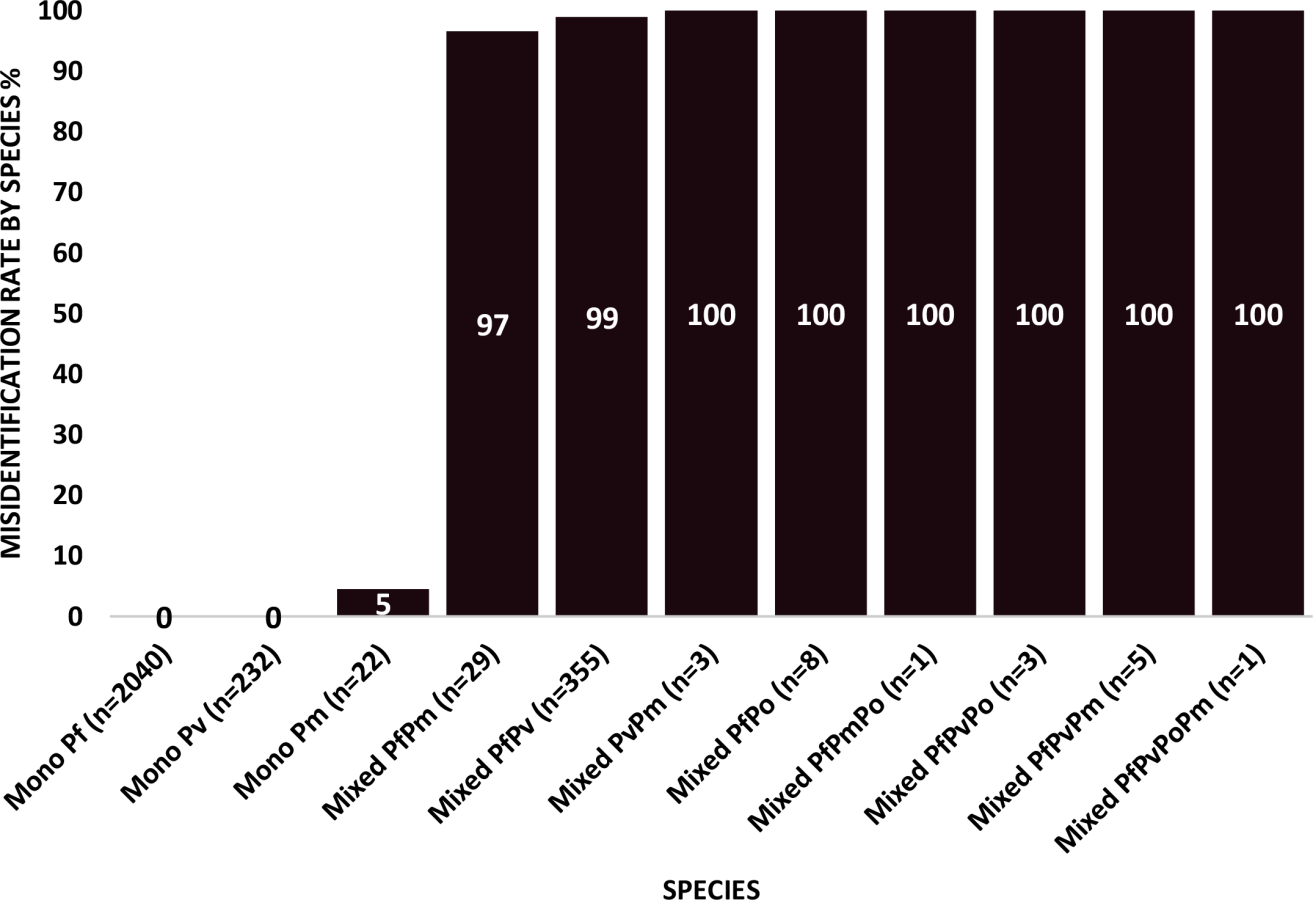
Contribution of *Plasmodium* species to microscopy misidentification. The figure presents percentage of misidentification (by microscopy) of a particular species from confirmed positive cases (labelled as ‘n’) of that particular species detected by PCR calculated as per the formula: $=\frac{\text{total misidentified cases by microscopy }(\text{of a particular species})\;}{\text{total PCR confirmed cases }(\text{of that particular species})}*100$.

As expected, Pf and Pv were correctly identified by microscopy but only when present as mono-infections (**Table 1**, **Figure 3**, **Supplementary Table S1**) and not as mixed-infections with other *Plasmodium* species wherein one of the species was found missing by microscopy. As shown in **Supplementary Table S1**, microscopy-false positives are consistent in Pf and Pv mono-infections across all studies, whereas microscopy-false negatives are restricted to almost all mixed-species infections, including PfPv. **Figure 3** illustrates the situation better by plotting species-specific misidentified cases out of total positive cases (by microscopy) of that species. The bars represent species-specific misidentification rate out of total positive cases of that particular species. As expected, microscopy did not misidentify any of the Pf (0/2040) or Pv (0/232) mono-infections to some other species or their combinations. Although, just one mono Pm case (5%; 1/22) was reported to be misidentified by microscopy. In contrast, the picture is completely reversed with the mixed infections (misidentification rate >95%). Microscopy misidentified 99% (351/355) of PfPv mixed infections followed by 97% (28/29) of PfPm mixed infections. Aside from that, the misidentification rate for mixed cases of PvPm (3/3), PfPo (8/8), PfPmPo (1/1), PfPvPm (5/5) and PfPvPoPm (1/1) by microscopy was 100%.

**Figure 4:**
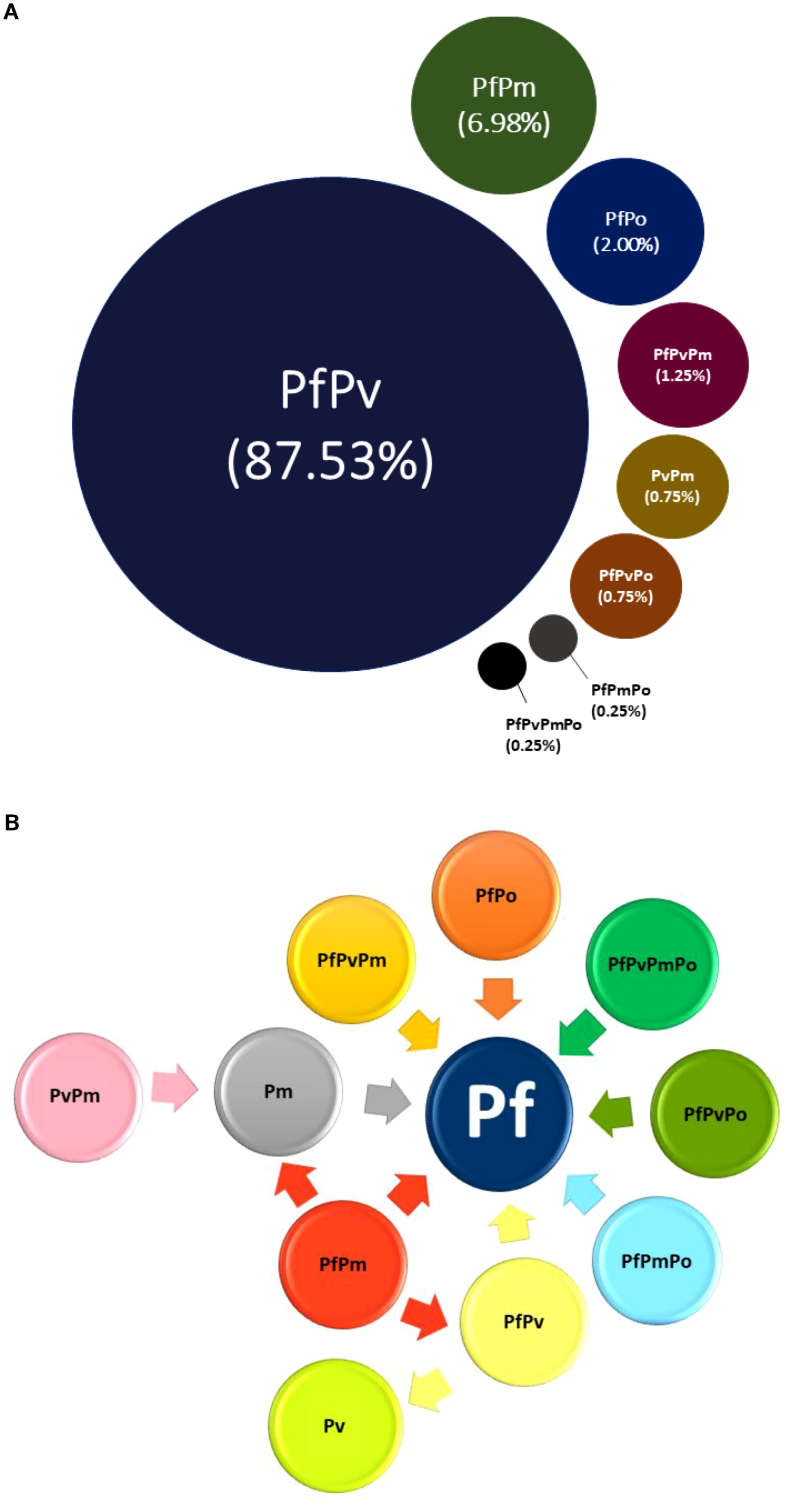
Misidentification of different *Plasmodium* species by microscopy. The top figure **(a)** shows the proportion (%) of different *Plasmodium* species and their combinations which were reportedly misidentified by microscopy out of total 401 samples which had misidentified species. The bottom figure **(b)** shows the dynamics of misidentification in terms of which species (or species combination) was misidentified into which different species or species combination. The arrow heads represent microscopy outcome, whereas the arrow tails represent PCR outcome from different reports. The triangular side of the arrow indicates the arrowhead, while the opposite end represents the tail.

**Figure 4a** and **Table 1** display the contribution of different species or their combinations (proportion; %) to the overall number of misidentified cases detected by microscopy. Out of total misidentified cases (n=401) irrespective of species, 88% (351/401) were mixed PfPv infections and 7% (28/401) were PfPm mixed infections showing a significant failure of a microscopist. Rest of the mixed infections that were misclassified constituted between 0.2 to 2% of the total misidentified infections. The most intriguing finding was that almost all mixed *Plasmodium* species infections (except PvPm) and Pm mono infection were misdiagnosed as Pf infection (either mono infection or mixed infection with other species) by microscopy, suggesting that Pf is the most familiar species to a microscopist. Surprisingly, PvPm mixed infection was misidentified as Pm mono infection. In addition, since the detailed breakup was not available in the studies, it was not possible to identify the proportion of PfPv and PfPm mixed infections that were misclassified into Pf and Pv and Pf, Pm and PfPv, respectively (**Table 1**). **Figure 4b** further highlights the issue that *Plasmodium* mixed-infections contribute the most to misidentification by microscopy and most of these are misidentified as *P. falciparum* infections, thus overestimating its burden.

## Discussion

Accurate diagnosis of malaria and its radical cure is crucial for global malaria control. This study revealed that when a non-Pf species coexists with Pf as mixed-infection, it is misidentified as *P. falciparum* by microscopy in >95% of the cases, indicating a significant failure in reporting species-specific prevalence (**Table 1**, **Figure 4b**). Importantly, the results indicate that the microscopists were able to differentiate Pf and Pv when present independently but fail to identify them together when present as mixed infections and instead tend to report them as mono-infections of one of the two species. There might be multiple reasons behind this observation. Although, it is possible that the other misidentified species present with Pf in mixed-infection were below the microscopy LoD, this cannot be true to all misidentified cases. In addition, it is difficult to accept that issues related to the sensitivity of microscopy are always associated with non-falciparum species. Similar findings were observed during a malaria microscopy remedial course conducted for ten days in Kenya and Ghana wherein misidentification was frequent with non-falciparum species (Obare et al., 2013). Microscopy has its own challenges at species-level diagnosis both in terms of the technology (threshold; LoD) and the microscopist (training) which are represented in the graphical abstract. In addition, issues such as negligence in reporting to maintain narrow turnaround time, recognition bias due to predominant regional prevalence of one species over the other, ‘unsaid’ reporting guidelines to report the most common species, etc.

Another possibility that could exist is the morphological similarities between different *Plasmodium* spp. making misidentification quite possible. Late stages (trophozoites, schizonts, and gametocytes) of *P. knowlesi* and *P. malariae* appear morphologically similar on blood smear. Immature trophozoites (ring forms) of *P. knowlesi* morphologically resemble those of *P. falciparum* having double chromatin dots (Lee et al., 2009). Both *P. ovale* and *P. vivax* infect immature erythrocytes, and their infected cells have amoeboid trophozoites. A multi-country meta-analysis was done to rule out Po misidentified as Pv and found that 11% of Po cases were misidentified as Pv in routine diagnosis (Kotepui et al., 2020). Another multi-country systematic review conducted to quantify the misidentification rate of Pk as Pm and reported 57% pooled prevalence (Mahittikorn et al., 2021). These results suggest that molecular assays are needed to reveal the true species-specific load and determine whether or not the species-specific parasite load qualifies as a sub-microscopic infection, provided that certain precautions are taken and their limits are taken into account (**Box 1**) (Deora et al., 2021; Deora and Sinha, 2022; WHO guidelines for malaria, 16 October 2023, 2023).

Box 1 **The box reframes two common probabilities of a false microscopy and PCR pair results with a new and detailed perspective. It also suggests recommendations for obtaining more factual conclusions from the false positive and false negative outcomes that might otherwise be frequently misinterpreted.**

New perspectives on false microscopy result

Microscopy False Positive Results:

Recommended action: perform nPCR for all *Plasmodium* spp. (pan-Plasmodium nPCR)

Probabilities:

(a) Actual ‘False Positive’: If pan-Plasmodium PCR yields a negative result, the sample is actually negative (eg. microscopy tells mono Pf but pan-Plasmodium PCR negative)

(b) Misidentified species: If pan-Plasmodium PCR yields negative result for the species identified by microscopy but positive for another species, the species is said to be misidentified (eg. microscopy suggests mono-Pf but pan-Plasmodium PCR confirms mono-Pv)

Microscopy False Negative Results:

Recommended action: perform qPCR for *Plasmodium* genus for determining parasite load

Probabilities:

(a) Sub-microscopic infections (SMI): If *Plasmodium* genus load is below the LoD of microscopy

If *Plasmodium* genus load is above the LoD of microscopy - perform qPCR for all *Plasmodium* species for determining species-specific parasite load

(b) Species-specific SMI: If *Plasmodium* species-specific load of all identified species is independently below the LoD of microscopy

(c) Actual ‘False Negative’: If *Plasmodium* species-specific load of any of the identified species is independently above the LoD of microscopy (microscopist’s incompetency or other technical issues)

Evidence exists that raise such issues in piecemeal indicating poor microscopy-based diagnosis and highlighting the need for trained and experienced microscopists in field settings (Mukadi et al., 2016; Mayengue et al., 2018).

The other important, but often neglected, issue is probing the reasons for a false positive (FP) microscopy result as found in this study where 7 microscopy positive samples turned out to be negative by PCR. These could be actually FP wherein microscopists identified some other hemoprotozoan (like Babesia or Theileria) as Plasmodium spp. or reported an artefact as a Plasmodium parasite. On the other hand, it is important to note that PCRs may provide a positive result due to presence of circulating dead parasites (not reported in microscopy) or parasite DNA after successful elimination of a natural infection. This could also be due to an erroneous PCR stemming from a contamination.

As countries approach malaria elimination with majority of cases geographically restricted to certain limited number of foci, timely and accurately detecting each and every *Plasmodium-*infected individual is of paramount significance. Left untreated, such infections may lead to sustenance and a gradual build-up of an infectious reservoir putting elimination at constant risk (Ouédraogo et al., 2009; Okell et al., 2012; Malaria surveillance, monitoring & evaluation: a reference manual, 2018). In this context, realizing the dependency of malaria control programs on microscopy, it is worthy to note that the major limitations with microscopy are its sensitivity and specificity which are driven by the microscopist competence and the technology *per se*. Not all microscopists have the same level of training and/or experience and therefore the LoD of microscopy ranges between 10 and 100 parasites/µL of blood. This lack of uniform competence also compromises the specificity of microscopy in terms of identifying the parasite species correctly leading to misidentification of parasite species. The combined effect of a compromised diagnostic accuracy may thus reflect in an over estimation of SMI (False SMI) in a region. In the context of species-specific diagnosis, this might under estimate the burden of certain “cryptic” parasite species which were not known to exist or exist as a rare species and this may have many downstream clinical and therapeutic ramifications (Chaturvedi et al., 2020).

### Challenges and suggested analytical approach

As an identified challenge, we would like to revisit the concept of false positive (FP) and false negative (FN) microscopy diagnosis for malaria because it is frequently misinterpreted when it comes to a falsely identified species within the *Plasmodium* genus. For example, if PCR detects an infection as mono Pf but the same is reported as mono Pv infection by microscopy, it becomes a FP microscopy diagnosis for Pv and at the same time a FN diagnosis for Pf.

### Possible misinterpretation of discordant microscopy results

Considering PCR as diagnostic gold standard, the discordant (FN & FP) microscopy results demand special attention as these generate outcomes that may be wrongly interpreted. We hereby suggest two interpretations of a microscopy FN result using an ultrasensitive qPCR (**Box 1**). As qPCR is able to estimate the parasite density, it will facilitate further classification of microscopy FN result into infections with parasite load below the LoD of microscopy (SMIs) and actual FN (parasite load above the LoD of microscopy but microscopist failed to detect parasite) and these are the instances where specific remedial interventions could be targeted. Similarly, microscopy FP results can be refined by using a pan-*Plasmodium* nested PCR into actual FP and species misidentification where both the scenarios would demand remedial training of concerned microscopists. It is also pertinent to say here that such granularity and factuality may be deciphered when each microscopy and PCR sample pair is analyzed one-to-one (proposed below) and not collectively as total number of samples (crude or overall analysis).

**Figure 5:**
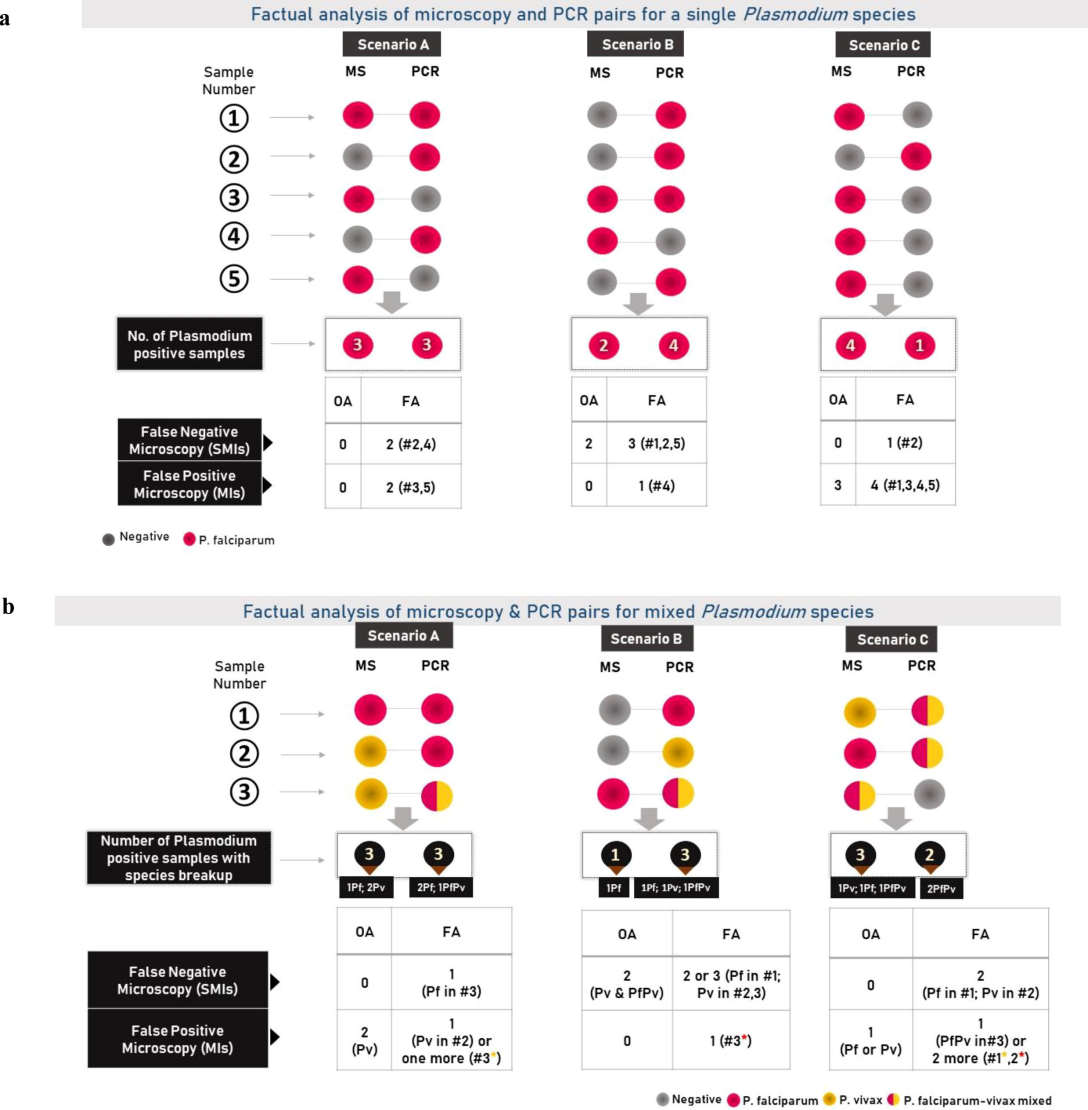
**(a)** Differential interpretations from overall (OA) and factual (one-to-one outcome; FA) analyses of microscopy and PCR paired samples results revealing various pitfalls in identification of false negative (by microscopy) or sub-microscopic infections. The figure depicts how interpreting the number of false negative or sub-microscopic infections from an overall analysis vs one-to-one analysis might lead to erroneous interpretation of results. Presented here are 3 possible and different scenarios (A–C) wherein the microscopy (MS) and PCR results of 5 independent samples (1-5) are mentioned. The pink and grey circles denote positive and negative results by the two methods, respectively. For the ease of interpretation, a positive result by both MS and PCR is assumed to be of the same *Plasmodium* species, i.e. *P. falciparum*. FN (false negative) and FP (false positive) are used to represent sub-microscopic infections and misidentified species, respectively. The comparative detailed interpretation from overall (OA) and factual (FA) analyses is also described. **(b)** Depiction of the complexity associated with mixed *Plasmodium* species in interpreting false negative (by microscopy) or sub-microscopic infections from an overall sample analysis (OA) versus factual (one-to-one sample) analysis (FA). Three different scenarios (A–C) are presented here, each with the Microscopy (MS) and PCR results of three independent samples (1-3). The pink, yellow, and grey circles represent the Pf, Pv, and negative results obtained by the two methods, respectively. Samples infected with both Pf and Pv are represented by circles that contain both pink and yellow. FN (false negative) and FP (false positive) denote sub-microscopic and misidentified infections, respectively. The comparative detailed interpretation from overall (OA) and factual (FA) analyses is also described. Samples with yellow asterisk show a possibility that Pf may have been misidentified as Pv, although Pv was also detected by PCR in that particular sample as a co-infecting species with Pf. Similarly, samples with orange asterisk demonstrate the opposite.

It is important to distinguish between overall analysis and one-to-one (paired) analysis of MS and PCR results. Overall analysis does not compare the results of the same individual samples; it only reports the total number of positives detected by MS and PCR, regardless of species. This can lead to misleading conclusions. For example, MS may record 3 samples as Pf, while PCR identifies them as mixed Pf and Pv. In this situation, the overall concordance appears to be 100%, but at the species level all three samples are actually discordant (detailed in **Figures 5a, b**). In contrast, one-to-one paired analysis compares the MS and PCR results of each specific sample. This provides a true picture of concordance and discordance because the outcome of each sample is directly matched between the two methods.

### Proposed factual (one-to-one) analysis approach

Crude/overall analysis of microscopy-PCR sample pairs tends to impart errors in actionable information interpreted from such data analysis as exemplified in **Figure 5a**. The figure which uses Pf as an example, puts forth three hypothetical scenarios having 5 such sample pairs in each scenario.

With respect to the number of FN or SMIs, the discrepancy in overall versus one-to-one (factual) analysis is evident in scenarios A (0 vs 2), B (2 vs 3) and C (0 vs 1) showing that the overall analysis always tends to underestimate the FN or possible sub-microscopic infections. The same is true for FP microscopy results as depicted in **Figure 5a**.

The interpretation becomes very critical while taking a corrective action as no corrective action is apparently needed for scenario A whereas wrong corrective measures may be adopted from scenarios B and C, if the overall analysis was followed. This becomes much more important as the remedies depend on the type of error – SMIs might be addressed by rectifying issues related to microscopy (missing the parasite) unless the parasite load is below the LoD of microscopy and a FP diagnosis might have roots in misidentifying a *Plasmodium* species or detecting an artefact as a parasite.

The issues discussed above become even more complex when species-specific SMIs are investigated (**Figure 5b**). At species level, mixed *Plasmodium* species infections provide distinct challenges for microscopy as the relative burden of each species remains unknown and one or more species load may be below the LoD of microscopy (SMI). In such situations, mixed-species infections are usually misdiagnosed as mono-species infections, resulting in a misleading diagnosis and treatment. Even with microscopically detectable burden, to maintain the turnaround time of diagnosis, the following issues might lead to incorrect diagnosis – ceasing to read the optimum number of fields as soon as one of the two or more species is identified and parasite burden estimated, relying on thick film for both detection and identification, using the blood smear made for full blood count to detect the parasites instead of making a dedicated stained peripheral blood smear for malaria, etc. In the absence of independent sample pairwise data from published reports (as suggested here), the true picture remains under cover. The species-specific SMI data is critical for malaria control program not only to identify the precise training needs but also to know if any specific *Plasmodium* species diagnosis is neglected thus leading to the build-up of a reservoir of infected people, which under suitable conditions, may lead to a sudden outbreak.

Despite the existing provisions for microscopy quality assurance (Internal Quality Control and External Quality Assessment Scheme) for continuous improvement in diagnostic accuracy of malaria microscopy in India, it has been difficult to sustain the efforts owing to operational, logistic and technical challenges (National Vector Borne Diseases Control Programme (NVBDCP), 2007). In addition, high quality malaria slide banks, an effective supply chain for equipment and consumables and timely recruitment against vacant posts are essential to ensure and sustain quality malaria microscopy.

With an increasing access to good speed internet and ‘smart’ mobile phones, both online and offline e-trainings may be easily imparted to all microscopists and their competence assessed at frequent intervals. Use of online trainings and educational games (Ozcan, 2014; Feng and Ozcan, 2015) and offline resources such as the Worldwide E-Learning Course on Malaria Microscopy (Amref Health Africa’s Institute of Capacity Development) may be promoted. In addition, development and use of digital microscopy and robust artificial intelligence-based approaches in malaria microscopy and strengthening WHO-recommended malaria slide banks could also offer a new vista in improving the outcomes of continuous microscopy trainings (Beck, 2022; Nema et al., 2022).

### Limitations of the study

The database for the current study was restricted to *Plasmodium* mixed-infection, but the authors recommend that more studies be conducted on *Plasmodium* mono-infections misidentification (by microscopy) to obtain a more complete picture. Because, in the case of mixed-infections that are misidentified as mono-infections, one species may be below the microscopy detection threshold, but this can be avoided in the case of mono-infections. In addition, because the included reports lack information on the expertise level of microscopists, the authors are unable to identify whether an expert microscopist was involved or not. It also lacks studies involving Pk because they were omitted from the database owing to insufficient details. It has been noted that most PCR-based studies included in the analyses did not attempt detecting non-falciparum species particularly *P. knowlesi*, *P. ovalecurtisi* and *P. ovalewallikeri*.

## Conclusion

*Plasmodium* misidentification by microscopy, coupled with the problem of SMI, are recognized yet under-reported problems in malaria-troubled countries. Both these attributes are the results of FP and FN microscopy-based malaria diagnosis, respectively, and have the potential to building up of undetected species-specific *Plasmodium* reservoirs that have a transmission potential and therefore thwart malaria elimination. Majority of published literature that reports misidentification and SMIs includes research not targeted specifically for quantifying misidentification and SMIs and report the same as a secondary or even a tertiary outcome. Those that do mention misidentification and SMIs often tend to report them in a flawed manner by adopting the summative analysis of microscopy-PCR paired results as shown here. The proposed one-to-one factual analyses of the microscopy-PCR paired samples may help in deciphering the misidentification and SMI data in a more granular way generating targeted actionable information for the program managers.

Further, identifying the states or specific locations with the highest rates of misidentification is essential for directing targeted support and corrective measures where they are most needed.

Finally, this research suggest that we might be overestimating the burden of *P. falciparum*, potentially misdirecting scarce elimination resources, while at the same time, underestimating non-falciparum species.

## Data Availability

The original contributions presented in the study are included in the article/**Supplementary Material**. Further inquiries can be directed to the corresponding author/s.
