# Supplementary material for: Plasmodium mono and mixed-infections in India: a tale of see-saw in species misidentification
Source: Front Cell Infect Microbiol. 2026 Jan 2;15:1695062. doi: 10.3389/fcimb.2025.1695062 (PMC12808463; doi:10.3389/fcimb.2025.1695062)
Supplement: Supplementary file 1 [file Table1.docx]

Table S1: The table displays misidentification (species-wise) data (number of cases detected by Microscopy and PCR) from 11 states derived from extracted reports, with the top row indicating the year of data collection. In the second row, states were arranged alphabetically & then according to year of data-collection in ascending order. MS: Microscopy, PCR: Polymerase Chain Reaction, AP: Arunachal Pradesh, AS: Assam, CH: Chhattisgarh, GUJ: Gujarat, JH: Jharkhand, KA: Karnataka, MP: Madhya Pradesh, MH: Maharashtra, OD: Odisha, RJ: Rajasthan, TR: Tripura

| **Study** | **Data collection site** | **Year of data collection** | **Microscopically positive samples** | **Samples correctly diagnosed by microscopy (concordant with PCR)** | **Samples misidentified by microscopy (discordant with PCR)** | **PCR result of misidentified samples** |
| --- | --- | --- | --- | --- | --- | --- |
|  |  |  | **N** | **N (species)** | **N (species)** | **N (species)** |
| Mohapatra et al. 2008^16^ **(##++)** | Arunachal Pradesh | 2005 | 9 | 8 (7-Pm; 1-PfPm) | 1 (Pm) | 1 (PvPm) |
| Dhiman et al. 2013^17^ (**#++)** | Assam | 2011 | 58 | 52 (48-Pf; 4-PfPv) | 6 (5-Pf; 1-PfPv) | 6 (1-Pm; 3-PfPm; 2-Neg) |
| Krishna et al. 2015^18^ **(#+)** | Gujarat | 2014 | 97 | 87 (Pf) | 10 (Pf) | 10 (8-PfPv; 2-PfPm) |
| Krishna et al. 2015^18^ **(#+)** | Jharkhand | 2014 | 216 | 158 (Pf) | 58 (Pf) | 58 (55-PfPv; 2-PfPo; 1-PfPmPo) |
| Krishna et al. 2015^18^ **(#+)** | Maharashtra | 2014 | 234 | 211 (Pf) | 23 (Pf) | 23 (21-PfPv; 2-PfPm) |
| Krishna et al. 2015^18^ **(#+)** | Odisha | 2014 | 267 | 219 (Pf) | 48 (Pf) | 48 (40-PfPv; 6-PfPm; 2-PfPo) |
| Krishna et al. 2015^18^ **(#+)** | Rajasthan | 2014 | 140 | 112 (Pf) | 28 (Pf) | 28 (26-PfPv; 2-PfPm) |
| Krishna et al. 2015^18^ **(#+)** | Tripura | 2014 | 127 | 107 (Pf) | 20 (Pf) | 20 (19-PfPv; 1-PfPm) |
| Bharti et al. 2013^19^ **(#++)** | Madhya Pradesh | 2012 | 22 | 14 (Pm) | 8 (Pm) | 8 (3-PmPf; 2-PmPv; 3-Neg) |
| Krishna et al. 2015^18^ **(#+)** | Madhya Pradesh | 2014 | 226 | 174 (Pf) | 52 (Pf) | 52 (47-PfPv; 4-PmPf; 1-PoPf) |
| Rishikesh et al. 2015^20^ **(##+)** | Karnataka | 2012-14 | 124 | 116 (Pv) | 8 (Pv) | 8 (PfPv) |
| Saravu et al. 2016^21^ **(##+)** | Karnataka | 2012-15 | 161 | 116 (Pv) | 45 (Pv) | 45 (PfPv) |
| Singh et al. 2013^22^ **(#+)** | Chhattisgarh | 2010 | 256 | 253 (Pf) | 3 (Pf) | 3 (2-PoPf; 1-PoPfPv) |
| Chaturvedi et al. 2015^23^ **(#+)** | Chhattisgarh | 2013-14 | 200 | 199 (Pf) | 1 (Pf) | 1 (PoPfPv) |
| Krishna et al. 2015^18^ **(#+)** | Chhattisgarh | 2014 | 214 | 188 (Pf) | 26 (Pf) | 26 (23-PfPv; 2-PmPf; 1-PoPf) |
| Krishna et al. 2017^24^ **(#+)** | Chhattisgarh | 2015 | 355 | 284 (Pf) | 71 (Pf) | 71 (59-PfPv; 5-PfPvPm; 3-PfPm; 1-PoPfPv; 1-PoPfPvPm; 2-Neg) |
|  |  | **Total** | **2706** | **2298** | **408** |  |

**Table S2: Detailed description of data analyzed from all nine included reports.** The table represents concordant and discordant results (species-wise) of microscopy and PCR. Additional information: Except for one study (Bharti et al. 2013), none of them had enough information to label their microscopists as "Experts". Further, the type of study design was marked as (##) Longitudinal, (#) Cross-sectional, (++) Active & (+) Passive. All the studies included in this analysis used a common nested PCR approach.
